# Diagnosing atmospheric communication of a sealed monitor chamber

**DOI:** 10.1002/acm2.12975

**Published:** 2020-07-10

**Authors:** Travis J. McCaw, Brendan A. Barraclough, Maxwell Belanger, Abigail Besemer, David A. P. Dunkerley, Zacariah E. Labby

**Affiliations:** ^1^ Department of Human Oncology University of Wisconsin Madison WI USA; ^2^ Department of Radiation Oncology University of Nebraska Medical Center Omaha NE USA; ^3^ Department of Radiation Oncology University of Iowa Iowa City IA USA

**Keywords:** ionization chamber dosimetry, quality assurance, statistical process control

## Abstract

Daily output variations of up to ±2% were observed for a protracted time on a Varian TrueBeam® STx; these output variations were hypothesized to be the result of atmospheric communication of the sealed monitor chamber. Daily changes in output relative to baseline, measured with an ionization chamber array (DQA3) and the amorphous silicon flat panel detector (IDU) on the TrueBeam®, were compared with daily temperature‐pressure corrections (*P*_TP_) determined from sensors within the DQA3. Output measurements were performed using a Farmer® ionization chamber over a 5‐hour period, during which there was controlled variation in the monitor chamber temperature. The root mean square difference between percentage output change from baseline measured with the DQA3 and IDU was 0.50% over all measurements. Over a 7‐month retrospective review of daily changes in output and *P*_TP_, weak correlation (*R* ^2^ = 0.30) was observed between output and *P*_TP_ for the first 5 months; for the final 2 months, daily output changes were linearly correlated with changes in *P*_TP_, with a slope of 0.84 (*R* ^2^ = 0.89). Ionization measurements corrected for ambient temperature and pressure during controlled heating and cooling of the monitor chamber differed from expected values for a sealed monitor chamber by up to 4.6%, but were consistent with expectation for an air‐communicating monitor chamber within uncertainty (1.3%, *k* = 2). Following replacement of the depressurized monitor chamber, there has been no correlation between daily percentage change in output and *P*_TP_ (*R* ^2^ = 0.09). The utility of control charts is demonstrated for earlier identification of changes in the sensitivity of a sealed monitor chamber.

**Abbreviations**
AAPM: American Association of Physicists in Medicine
DQA3: ionization chamber array
IDU: amorphous silicon flat panel detector
MPC: Machine Performance Check
*P*_TP_: temperature‐pressure correction

## 1 INTRODUCTION

Medical linear accelerators use ionization chambers positioned within the beam path to monitor the radiation fluence produced and terminate an irradiation when the programmed fluence has been delivered. The fluence measured by the monitor chambers is related to the dose delivered to a specified point for a reference set of irradiation conditions as defined by a clinical reference dosimetry protocol (e.g., American Association of Physicists in Medicine (AAPM) Task Group 51).^1^ Monitor chambers can either be sealed such that they contain a nominally constant mass of gas or open to the atmosphere such that the mass of gas within the chamber is dependent on ambient air temperature and pressure. TrueBeam® linear accelerators (Varian Medical Systems, Inc., Palo Alto, CA) use sealed monitor chambers to eliminate the need to correct measured ionization for atmospheric conditions.

The AAPM recommends that the constancy of dose per monitor unit be verified with daily measurements.^2^ From June to December 2017, the standard deviation in daily measurements of dose per monitor unit relative to baseline with an ionization chamber array (Daily QA™3, Sun Nuclear Corporation, Melbourne, FL) for the Varian TrueBeam® STx accelerator at our institution was 0.36%; between December 20, 2017 and February 23, 2018, the standard deviation in daily measurements of dose per monitor unit relative to baseline increased to 0.84%. This increased standard deviation in daily measurements of dose per monitor unit was determined to be the result of atmospheric communication of the sealed monitor chamber. While long‐term output trend analyses for linear accelerators with sealed monitor chambers have previously been reported,^3^, ^4^ atmospheric communication of a sealed monitor chamber has only been reported for an obsolete linear accelerator model with limited guidance on the process for diagnosis of atmospheric communication.^5^ The purpose of this work was to develop a process for the diagnosis of atmospheric communication of a sealed monitor chamber.

## 2 MATERIALS AND METHODS

To verify that the increased variation in daily measurements of dose per monitor unit with the DQA3 was not due to measurement error, independent measurements of the change in dose per monitor unit relative to baseline were made daily with the amorphous silicon flat panel detector (IDU) on the TrueBeam® STx using the beam constancy check of the TrueBeam® Machine Performance Check (MPC) application beginning on December 11, 2017. Consistency of MPC output constancy measurements with ionization chamber measurements has previously been demonstrated.^6^

The DQA3 has built‐in thermistors and pressure sensors to correct measurements for atmospheric conditions. A visual review of trended daily temperature‐pressure corrections (*P*_TP_) as determined by the DQA3 revealed that, by December 20, 2017, the daily variations in dose per monitor unit were consistent in magnitude with the variations in *P*_TP_, suggesting that the monitor chamber was communicating with the atmosphere.

To test for atmospheric communication of the monitor chamber, ionization measurements were performed using a PTW 23333 Farmer® ionization chamber (PTW, Freiburg, Germany) during controlled temperature variation in the monitor chamber. The Farmer® ionization chamber was positioned in a Solid Water® (Sun Nuclear Corporation, Melbourne, FL) phantom at a depth of 10 cm with 5 cm of backscatter. Ionization measurements for irradiations of 100 MU were completed using a PTW Unidos E electrometer over a 5‐h period during which the temperature of the monitor chamber was initially increased using a heat gun, then decreased using air conditioning and a fan. The temperatures of both the monitor chamber and the Solid Water® phantom were measured prior to each irradiation using a noncontact infrared thermometer. Each temperature measurement was repeated three times in succession across the visible surfaces of the monitor chamber and the Solid Water® phantom to provide an estimate of uncertainty due to temperature gradients and repeatability of the thermometer. All measurements were performed using the 6 MV beam energy; however, the increased variations in daily output measurements that motivated this investigation were observed for all energies.

**Fig. 1:**
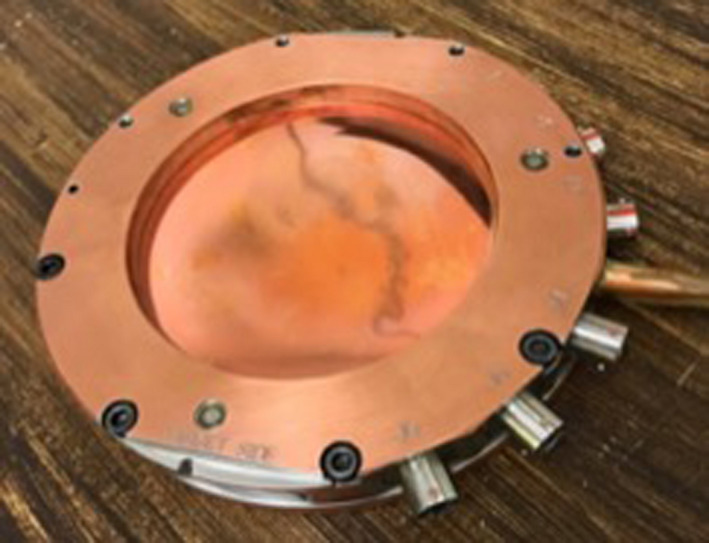
Photograph of the monitor chamber removed from the linear accelerator on 2/23/2018. The central film surface is concave, characteristic of a depressurized monitor chamber.

Changes in the ionization measurements corrected for ambient temperature and pressure were compared with expectation assuming (a) the monitor chamber was sealed and (b) the monitor chamber was air communicating. For the assumption of a sealed monitor chamber, the corrected ionization measurements should be constant. For an air‐communicating monitor chamber, the corrected ionization measurements should be directly proportional to changes in *P*_TP_ since a decrease in ambient air density (i.e., increase in *P*_TP_) requires a greater fluence at the monitor chamber to produce a given quantity of ionization within the monitor chamber (i.e., monitor unit). Based on the results of these measurements, the monitor chamber was replaced on February 23, 2018 and found to have a concave film surface characteristic of depressurization (Fig. 1).

## 3 RESULTS AND DISCUSSION

**Fig. 2:**
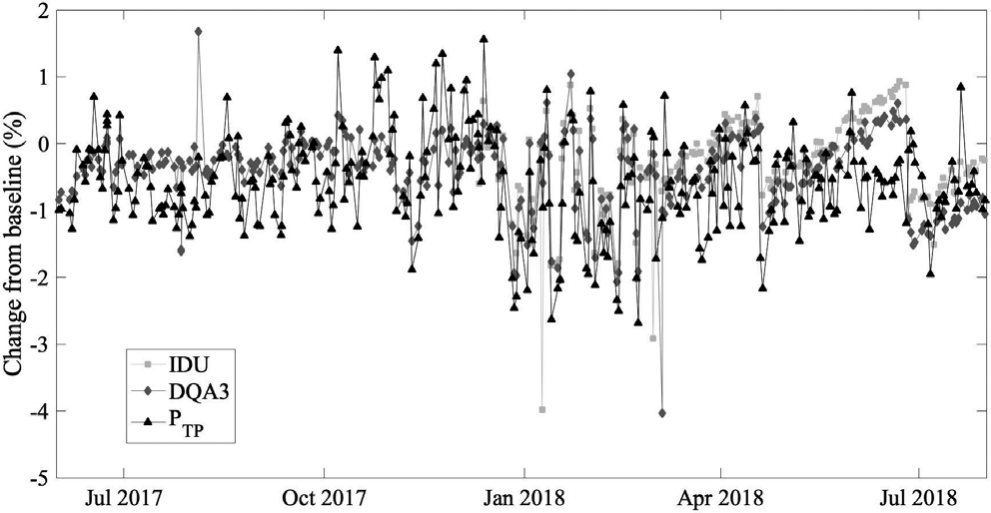
Percentage change in output from baseline measured daily using an ionization chamber array (DQA3) and an amorphous silicon flat panel detector (IDU). The output baseline for the flat panel detector was set on 12/10/2017, so there is no data prior to this date. The percentage change from the temperature‐pressure correction (*P*_TP_) at the time of baseline is also shown. The monitor chamber was replaced on 2/23/2018.

**Fig. 3:**
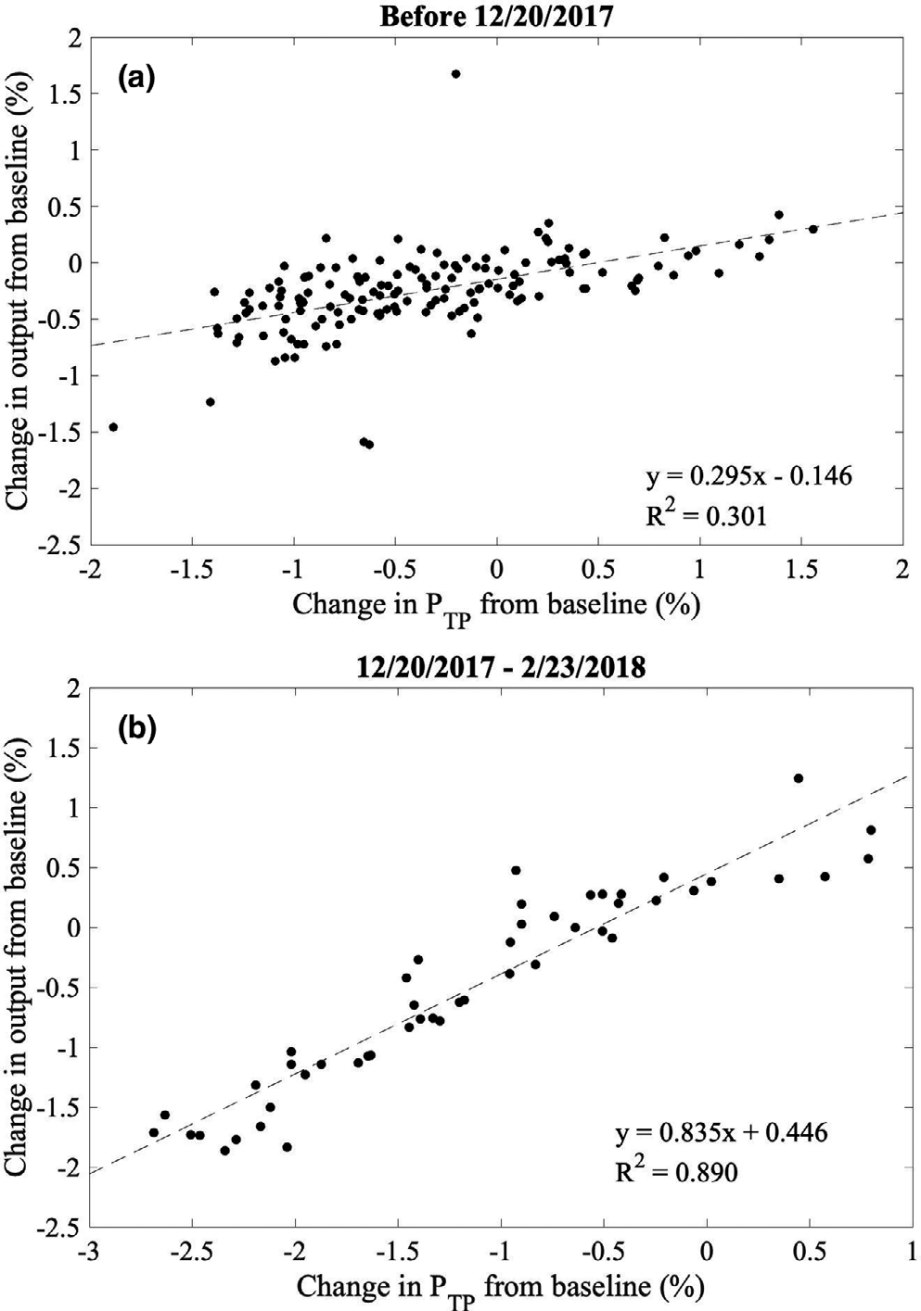
Percentage change in output from baseline measured daily using an ionization chamber array plotted versus the percentage change from the temperature‐pressure correction (*P*_TP_) at the time of baseline. Review of daily output and *P*_TP_ measurements suggests that the monitor chamber was communicating with the atmosphere by 12/20/2017; the monitor chamber was replaced on 2/23/2018.

The root mean square difference between percentage change in dose per monitor unit measured with the DQA3 and the IDU is 0.50% over all measurements prior to the replacement of the monitor chamber. This agreement between DQA3 and IDU measurements provides verification of the observed increase in the standard deviation of DQA3 measurements (from 0.36% to 0.84%) that motivated this investigation. Figure 2 shows the percentage change from baseline in the dose per monitor unit measured daily using the DQA3 and the IDU, as well as the percentage change from baseline in *P*_TP_ as measured by the DQA3. Percentage changes from baseline in daily measurements of dose per monitor unit and *P*_TP_ are weakly correlated prior to December 20, 2017 [Fig. 3(a)]. No correlation is expected between the DQA3 measurements and *P*_TP_ variation if the monitor chamber is sealed since the DQA3 measurements are corrected for *P*_TP_; the observed correlation is further discussed later in this section. Between December 20, 2017 and the replacement of the monitor chamber on February 23, 2018, DQA3 measurements are linearly correlated with atmospheric changes, with a slope of 0.84 [*R* ^2^ = 0.89, Fig. 3(b)].

**Fig. 4:**
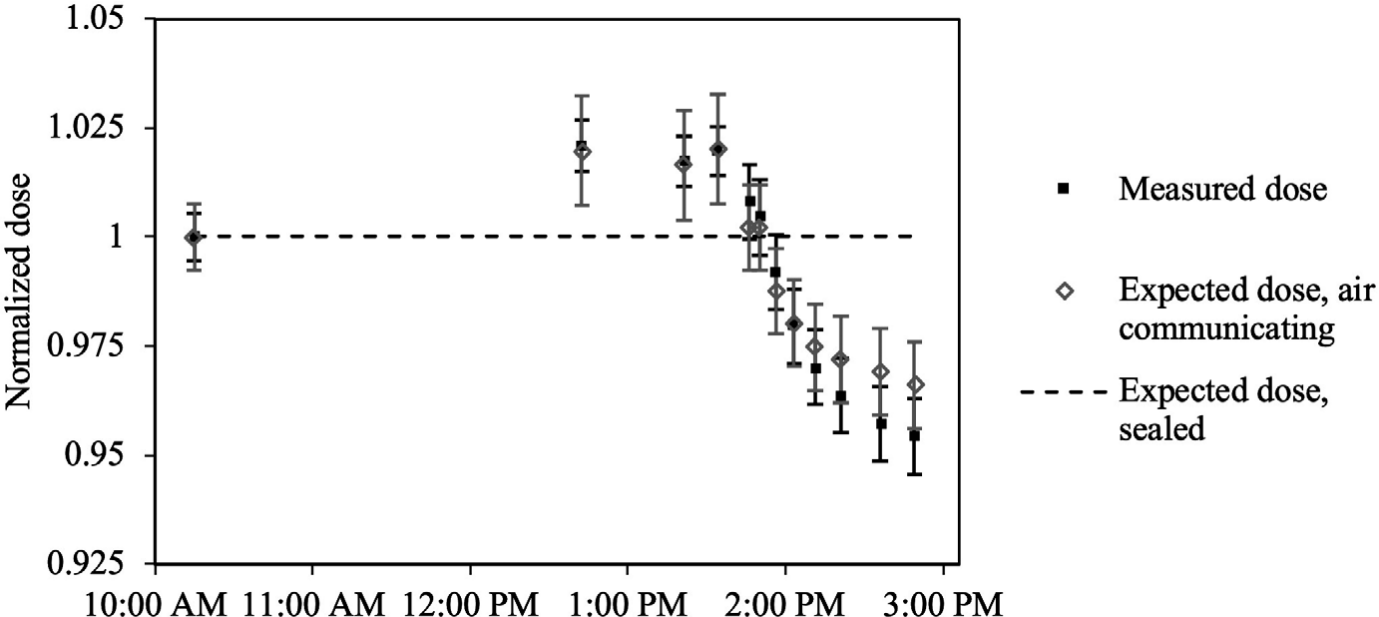
Relative change in dose measured with a Farmer® ionization chamber during controlled variation in the monitor chamber temperature over a 5‐h period. The monitor chamber was heated between 10:30 AM and 1:30 PM, then cooled starting at 1:30 PM. Expected changes in measured dose for hypotheses of a sealed monitor chamber and an air‐communicating monitor chamber are shown for comparison. Error bars show expanded (*k* = 2) overall uncertainty.

Measured changes in ionization corrected for ambient temperature and pressure during controlled temperature change in the monitor chamber are shown in Fig. 4, along with the changes in corrected ionization expected for both an air communicating and a sealed monitor chamber. The results in Fig. 4 are presented as relative dose normalized to the initial measurement since all other correction factors were constant for all measurements. The error bars in Fig. 4 for the expected dose for an air‐communicating monitor chamber give the expanded (*k* = 2) overall uncertainty determined by propagation of Type A uncertainty in measured temperature of the monitor chamber, while the error bars for measured dose give the expanded overall uncertainty determined by summation in quadrature of Type A uncertainty in measured ionization and Type B uncertainty in measured temperature due to temperature gradients between the surface of the Solid Water® phantom and at the depth of the Farmer® ionization chamber. There is a significant difference between measured and expected changes in dose for the assumption of a sealed monitor chamber, while the expected change in dose for the assumption of an air‐communicating monitor chamber agrees with measured changes in dose within uncertainty.

**Fig. 5:**
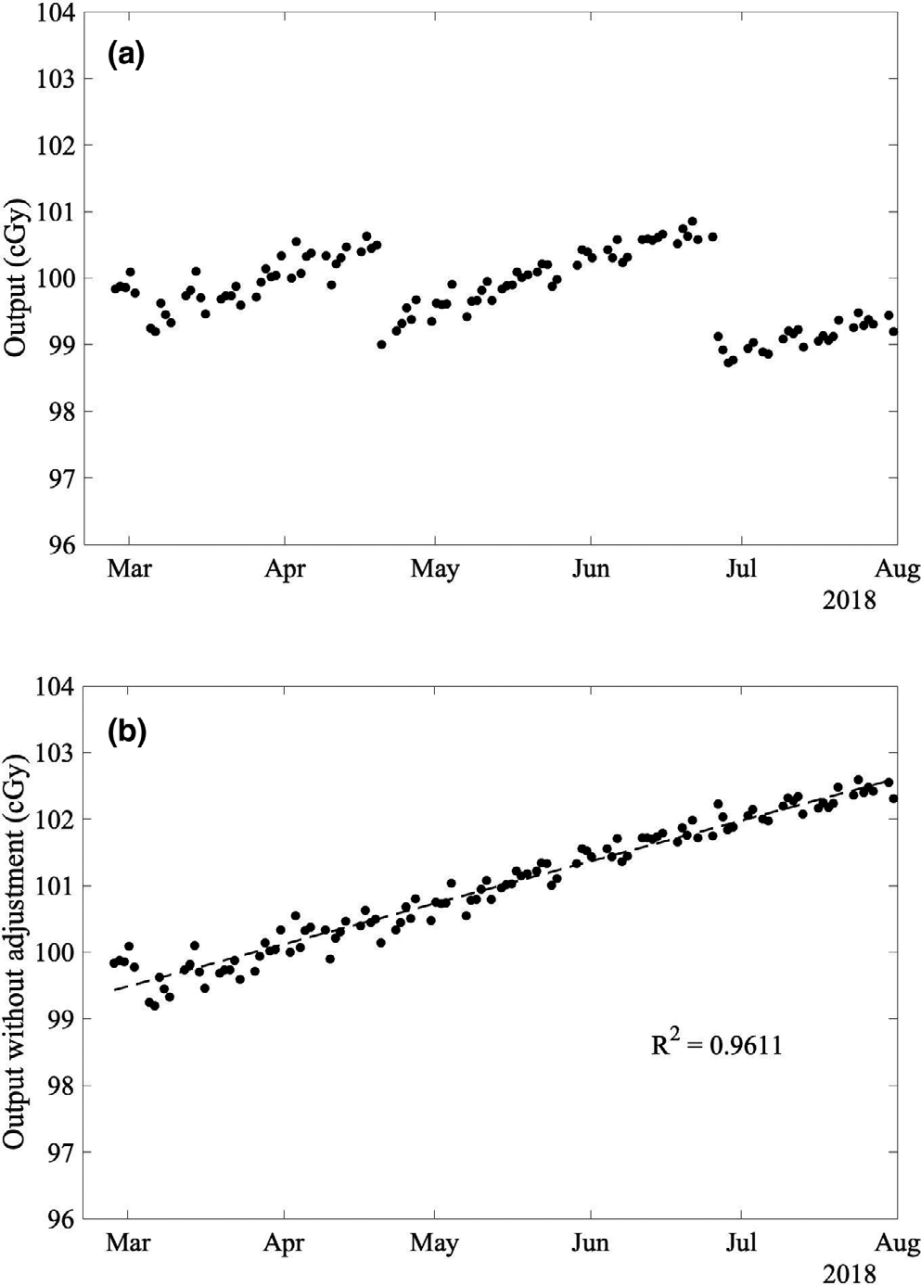
(a) Daily output measurements with the ionization chamber array (DQA3) following replacement of the monitor chamber. The discontinuities are consistent with calibration events. (b) Linear fit applied to adjusted daily output measurements with the effect of the calibration events removed.

**Fig. 6:**
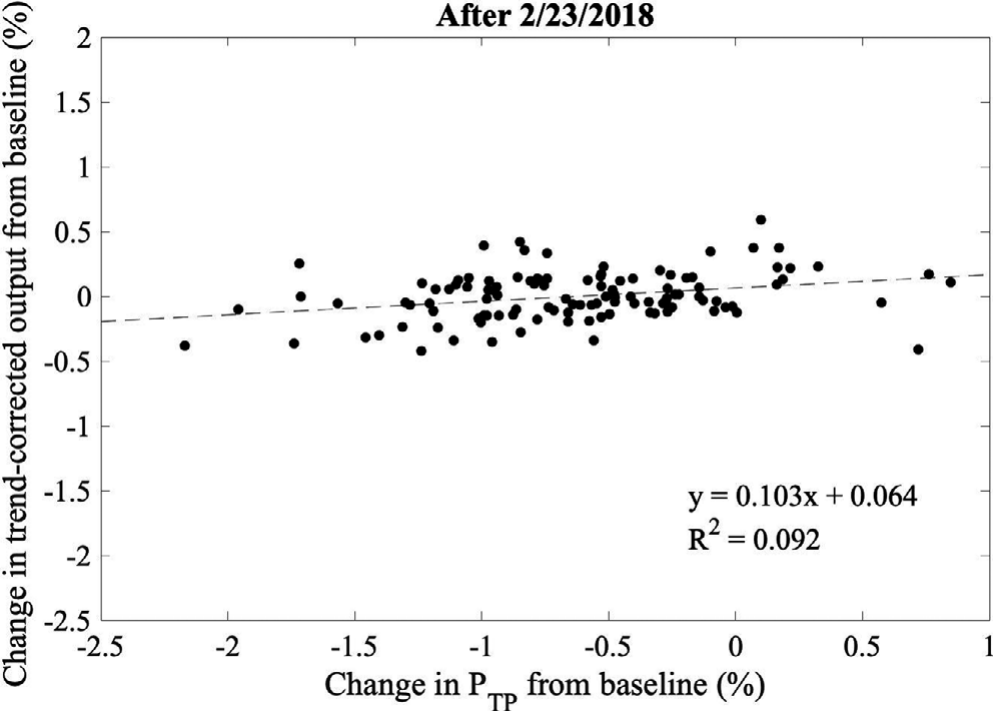
Percentage change in output from baseline measured daily using an ionization chamber array plotted versus the percentage change from the temperature‐pressure correction (*P*_TP_) at the time of baseline. The monitor chamber was replaced on 2/23/2018.

Following replacement of the monitor chamber on February 23, 2018, there has been a systematic increase in the measured output of 0.6%/month [Fig. 5(a)], with two output calibration events indicated by the discontinuities in the measurements. This increasing trend in output is consistent with the tendency of sealed monitor chambers to slowly lose sensitivity over time due to depressurization resulting from radiation‐induced reaction of oxygen within the monitor chamber with the Kapton windows.^7^ Similar increases in output have been reported by the manufacturer (approximately 1%/10^5^ monitor units),^7^ and also by multiple institutions during the commissioning of Varian TrueBeam® accelerators.^8^ The trend in output of an accelerator over a short (e.g., 6 month) time period has previously been modeled using a linear relation.^9^ The output measurements following the replacement of the monitor chamber were adjusted to remove the effect of the output calibrations, and linear least squares regression was used to model the increasing trend in output [Fig. 5(b)]. The output measurements were then corrected for the modeled trend to remove the systematic variation. Over the first 5 months of measurements since the replacement of the monitor chamber, there has been no correlation between trend‐corrected DQA3 measurements and atmospheric changes (Fig. 6), and the standard deviation in trend‐corrected DQA3 measurements has been reduced to 0.18%.

**Fig. 7:**
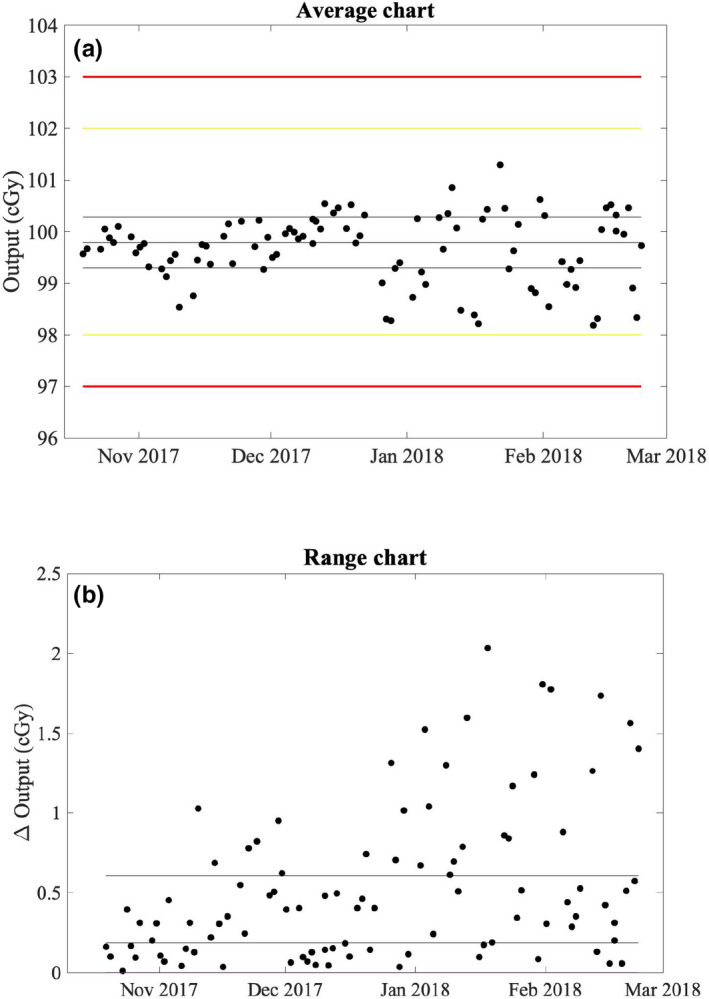
Average (a) and range (b) charts for the daily output measurements with the ionization chamber array (DQA3) before replacement of the monitor chamber using a subgroup size of one. Consecutive results in the average chart were used to calculate the range. Control limits indicated by the thin solid lines were calculated using the first 10 data points. The yellow and red lines indicate institutional limits for scheduled and immediate action, respectively.

**Fig. 8:**
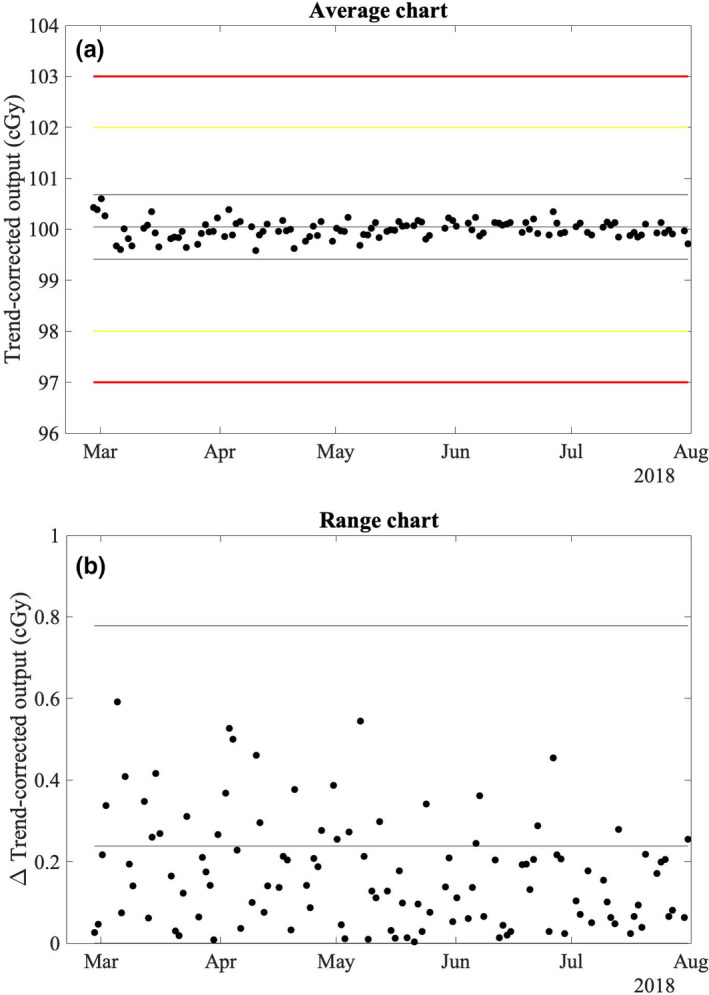
Average (a) and range (b) charts for the daily output measurements with the ionization chamber array (DQA3) after replacement of the monitor chamber using a subgroup size of one. Consecutive results in the average chart were used to calculate the range. Effects of output calibration events and the increasing trend in output have been removed. Control limits indicated by the thin solid lines were calculated using the first ten data points. The yellow and red lines indicate institutional limits for scheduled and immediate action, respectively

Figure 3(a) suggests that the monitor chamber may have been communicating with the atmosphere prior to December 20, 2017; however, no action was taken due primarily to continued constancy of daily output measurements within our institutional limits of ±2% for scheduled action. To facilitate more rapid diagnosis of changes in the response of a sealed monitor chamber, control charts were employed retrospectively following the methodology of Pawlicki et al.^10^ for daily output measurements. The average and range charts for daily output measurements prior to replacement of the monitor chamber are shown in Figs. 7(a) and 7(b), respectively. Control limits were calculated using 10 consecutive data points starting October 19, 2017, on which date a new DQA3 device was brought into clinical service. For this work a single data point outside of the control limits, indicated by the thin solid lines in Figs. 7(a) and 7(b), was considered nonrandom variation. Since the control limits for the average chart were less than half of our institutional limits for scheduled action, additional measures of nonrandom behavior (e.g., sequences of data points above or below the process mean) were not considered clinically relevant. Nonrandom variation in the mean and range of daily output measurements is observed as early as November 6, 2017, more than 6 weeks prior to the supposed start of atmospheric communication of the monitor chamber. The only explanation that could be identified for the results outside of the control limits was variation from baseline atmospheric conditions at the time of measurement in excess of that during the ten measurements used to generate the control limits. Therefore, the monitor chamber was likely communicating with the atmosphere at least as early as November 6, 2017. Measurements prior to October 19, 2017 were not considered in this analysis due to the systematic change introduced to the process with the replacement of the DQA3 device. Control limits were recalculated for the average and range charts of the daily output measurements corrected for the systematic output variation following the replacement of the monitor chamber [Figs. 8(a) and 8(b)], and no data points were outside of the updated control limits.

## 4 CONCLUSIONS

Increased daily variations in the dose per monitor unit measured on a Varian TrueBeam® STx were shown to be the result of atmospheric communication of the sealed monitor chamber. The measured variations in dose per monitor unit were confirmed with independent measurements using a separate detector. Ionization measurements acquired during controlled temperature variation in the monitor chamber confirmed atmospheric communication of an originally sealed chamber. Following replacement of the monitor chamber, there was no evidence of correlation between dose‐per‐monitor‐unit measurements and atmospheric conditions. The use of average and range charts to identify nonrandom variations in dose‐per‐monitor‐unit measurements outside of control limits was shown to be effective for earlier diagnosis of changes in the response of a sealed monitor chamber.

## CONFLICT OF INTEREST

No conflicts of interest.
